# Development of a Video Consultation Patient-Satisfaction Questionnaire (vCare-PSQ): A Cross-Sectional Explorative Study

**DOI:** 10.2196/58928

**Published:** 2024-08-02

**Authors:** Anders Larrabee Sonderlund, Tessa Quirina Bang Van Sas, Sonja Wehberg, Linda Huibers, Jesper Bo Nielsen, Jens Søndergaard, Elisabeth Assing Hvidt

**Affiliations:** 1 Research Unit of General Practice Institute of Public Health University of Southern Denmark Odense Denmark; 2 Research Unit of General Practice Aarhus Denmark

**Keywords:** video consultation, patient satisfaction, patient-physician relationship, telehealth, general practice, pilot-testing, COVID-19, SARS-CoV-2, pandemic, primary care, healthcare, health professional, health professionals, Danish, adult, adults, IT-literacy, methodological, vCare-PSQ, COSMIN

## Abstract

**Background:**

Since the COVID-19 pandemic, the use of video consultation (VC) in primary care has expanded considerably in many countries. VC and other telehealth formats are often touted as a solution to improved health care access, with numerous studies showing high satisfaction with this care format among health professionals and patients. However, operationalization and measurement of patient satisfaction with VC varies across studies and often lacks consideration of dynamic contextual factors (eg, convenience, ease-of-use, or privacy) and doctor-patient relational variables that may influence patient satisfaction.

**Objective:**

We aim to develop a comprehensive and evidence-based questionnaire for assessing patient satisfaction with VC in general practice.

**Methods:**

The vCare Patient-Satisfaction Questionnaire (the vCare-PSQ) was developed according to the COSMIN (Consensus-Based Standards for the Selection of Health Measurement Instruments) guidelines. To achieve our overall objective, we pursued three aims: (1) a validation analysis of an existing patient-satisfaction scale (the PS-14), (2) an assessment of extrinsic contextual factors that may impact patient satisfaction, and (3) an assessment of pertinent intrinsic and relational satisfaction correlates (eg, health anxiety, information technology literacy, trust in the general practitioner, or convenience). For validation purposes, the questionnaire was filled out by a convenience sample of 188 Danish adults who had attended at least 1 VC.

**Results:**

Our validation analysis of the PS-14 in a Danish population produced reliable results, indicating that the PS-14 is an appropriate measure of patient satisfaction with VC in Danish patient populations. Regressing situational and doctor-patient relational factors onto patient satisfaction further suggested that patient satisfaction is contingent on several factors not measured by the PS-14. These include information technology literacy and patient trust in the general practitioner, as well as several contextual pros and cons.

**Conclusions:**

Supplementing the PS-14 with dynamic measures of situational and doctor-patient relational factors may provide a more comprehensive understanding of patient satisfaction with VC. The vCare-PSQ may thus contribute to an enhanced methodological approach to assessing patient satisfaction with VC. We hope that the vCare-PSQ format may be useful for future research and implementation efforts regarding VC in a general practice setting.

## Introduction

### Background

The use of video consultation (VC) in general practice increased significantly in many countries during the COVID-19 pandemic [1]. This mode of remote health care delivery was implemented to ensure continued health care access while maintaining social distancing restrictions. Post the pandemic, however, the perceived value and clinical efficacy of VC compared to in-person consultations is unclear [1]. While studies indicate that VC might be a useful tool in efforts to optimize primary care efficacy and bandwidth, its large-scale feasibility is fundamentally contingent on the extent to which practitioners and patients are willing to use it [2]. To this point, emerging research has focused on patient satisfaction with VC as a key factor in its continued postpandemic expansion.

Existing quantitative and qualitative literature suggests that patient satisfaction with VC manifests in myriad ways and is subject to multiple factors. For example, several studies have operationalized satisfaction in terms of perceived convenience (eg, reduced travel and waiting times, lower travel costs, faster access, and practitioner-patient communication) [2-8], while other research has uncovered factors that may compromise satisfaction, including patient worry about subpar care (compared to in-person consultations) [3,9], concerns about effective symptom communication [3,9], medical mistrust and privacy issues [10,11], as well as lack of technical know-how [9,12,13]. Given these numerous correlates and manifestations of patient satisfaction, measuring this construct reliably and comprehensively represents a difficult task. Indeed, to date, most empirical quantitative assessments of patient satisfaction have relied on relatively one-dimensional self-report scales [2]. While these measures may provide face-value insight into the general individual-level experience of VC, they often fall short of assessing the intersecting range of relational (eg, doctor-patient relationship or online communication) [14], organizational (eg, making appointments or technology availability) [15], and even structural factors (eg, socio-economy, the digital divide, or rurality) [16,17] that likely underpin patient satisfaction. Given the diversity and intersectionality of patient populations and the contexts in which they are embedded, knowledge about these underlying factors that shape patient satisfaction represents important information about how VC might be better tailored and equitably implemented in various settings and health care systems [2]. In other words, these methodological shortcomings pose considerable constraints for the continued scientific evaluation of VC appropriateness, patient acceptability, and efficacious delivery.

### Rationale and Aims

The overall objective of this study is to develop a comprehensive and dynamic multidimensional questionnaire (the vCare Patient-Satisfaction Questionnaire [vCare-PSQ]) for evaluating patient satisfaction with VC. To achieve this objective, we pursue 3 specific aims. First, as the cornerstone of our questionnaire, we rely on an existing VC patient-satisfaction scale (PS-14). The PS-14 was validated in 1999 [18] and developed for gauging patient satisfaction with VC in an American prison population. To use it for our purposes, however, it is necessary to determine its appropriateness in not only a Danish and modern primary care setting, but also in the general population (as opposed to a prison population). Thus, aim 1 comprises a preliminary validity analysis of the PS-14 in a Danish sample. The validated PS-14 represents the core and standardized dimension of patient satisfaction in our questionnaire.

Next, we recognize that patient satisfaction is not necessarily stable across settings, but rather a dynamic construct that may be influenced by variable contextual factors (eg, available VC technology, time pressures, and awareness of a chronically swamped health care system). In aim 2, we, therefore, assess whether adding a dynamic component, which assesses contemporary and situational patient-reported pros and cons of VC, might complement the more static PS-14 and thus afford greater insight into the context-dependent determinants of patient satisfaction.

Finally, we argue that there are also interpersonal and intrinsic patient factors that underpin satisfaction with VC, and which extend beyond those measured in the PS-14. Thus, in aim 3 we examine additional intrinsic patient-general practitioner (GP) relational variables that may further enhance the PS-14 by facilitating a deeper understanding of the drivers of patient satisfaction. Specifically, based on previous evidence that IT literacy, health anxiety, and patient-doctor trust and familiarity are fundamental to patient satisfaction with health care in general, we assess the extent to which these factors also feed into patient satisfaction with VC in particular [11,19,20].

We hope that our conceptualization and empirical operationalization of patient satisfaction in these multidimensional terms (outlined in aims 1-3) may provide a jumping-off point for future research and facilitate a better and more comprehensive understanding of the core and contextual factors of satisfaction with remote care delivery.

## Methods

### vCare-PSQ Development

The vCare-PSQ was developed and validated according to COSMIN (Consensus-Based Standards for the Selection of Health Measurement Instruments) guidelines [21]. This comprised a rigorous process that included a comprehensive literature review of relevant validated questionnaires, cognitive interviews with patients who had participated in VCs, and pilot testing of the questionnaire by a panel of experts in primary and digital care. Specifically, and in line with aim 1, we first conducted a systematic review of the peer-reviewed literature to identify any relevant VC evaluation instruments. The search identified 14 measurement scales that were pertinent to the broader field of patient satisfaction with telehealth. Of these, 1—the PS-14 [18]—was specifically related to VC satisfaction and was retained for our study. Next, for aims 2 and 3, additional items (tapping patient-doctor relational aspects of VC [trust and familiarity], patient health anxiety and IT literacy, pros and cons of VC, or demographics) were included from a questionnaire that was recommended by an expert in the field of remote consultations. This was a nonvalidated questionnaire that was developed and used in recent (2018) research on VC in the United Kingdom (the ViCo Study) and translated into Danish [8,22]. Once a preliminary draft of the questionnaire for our study was complete, we conducted a face validation of the instrument based on cognitive interviews with 12 patients who had participated in VCs. The interviews were designed to further develop the questionnaire through critical deliberation and assessment of each item. Finally, the questionnaire was pilot-tested and evaluated by an expert panel of 18 GPs and research scientists.

### vCare-PSQ Measures

The variables included in the vCare-PSQ were based on the available evidence that indicated their relevance for patient satisfaction. These corresponded to our three aims and pertained to (1) general patient satisfaction with the VC format compared to in-person consultations; (2) contextual patient-reported pros and cons of VC, including for example technology-, sustainability-, location-, organization-related issues; and (3) key patient and patient-GP relational variables including quality of patient-GP relationship (familiarity and trust) health anxiety before a call, and IT literacy. We also included patient demographics for gender, age, education, and living status.

#### Aim 1: General Patient Satisfaction With VC Format

This variable was measured using the 14-item validated PS-14 scale by Mekhjian et al [18], which was originally designed for VC evaluation in prison populations. The 14-item instrument was forward-backward translated (English-Danish) according to World Health Organization guidelines [23] and adapted for our study. The scale included items on overall satisfaction with VC as well as satisfaction related specifically to technology, patient-GP communication, and VC compared to in-person consultations in terms of general satisfaction as well as satisfaction with the treatment received. Items were measured on 5-point Likert scales (1=strongly disagree, 5=strongly agree) and averaged to create a single summary score for patient satisfaction. Responses with more than 2 missing values were excluded, as were uniform responses of only “5” or “1” across all 14 items.

#### Aim 2: Contextual Patient-Reported Pros and Cons

Patient acceptability and convenience were measured using 2 categorical checklists of 10 possible pros and 10 possible cons, sourced from the ViCo Study and 12 semistructured interviews with patient users of VC. Perceived pros of VC related to reducing the patient burden on the primary care system, environmental sustainability, easier appointment scheduling and participation, less anxiety, time-saving, and less time off required to attend an appointment. Cons included the need for in-person follow-ups canceling out advantages of an initial VC, technology and communication issues, concerns about receiving subpar treatment (compared to in-person consultations), concerns about privacy during a call, poorer “bedside manner,” and virtual waiting room malfunctions.

#### Aim 3: Key Variables—Patient-GP Relationship Quality, Health Anxiety, and IT Literacy

##### Patient-GP Relationship Quality

Patient-GP relationship quality was operationalized using 2 items. One measured patient familiarity with their GP on a 4-point Likert scale (1=low familiarity, 4=high familiarity), and the other measured patient trust in their GP on a 4-point Likert scale (1=low trust and 4=high trust).

##### Health Anxiety Before VC

Health anxiety was assessed using a single-item measure that gauged the extent to which patients were worried about whether they had a serious illness before the VC. This item was measured on a 5-point Likert scale (0=low anxiety and 4=high anxiety).

##### IT Literacy

This variable measured whether participants felt more or less capable in terms of navigating IT. This was measured on a single-item 4-point Likert scale (1=low literacy and 4=high literacy).

#### Sociodemographic Participant Information

We collected sociodemographic information about age, gender (man, woman, or other), education (elementary school, vocational, high school, some college, bachelor’s degree, or master’s degree or higher), and living status (alone, partner, or with others). Living status was coded as a binary variable (single vs with others) and education was coded as a continuous variable (1=lowest and 6=highest).

### Ethical Considerations

This study was approved by the institutional review board at the University of Southern Denmark (11.497), the University of Southern Denmark Research Research Ethics Committee (21/71057), and was conducted per European Union General Data Protection Regulations. All participants were provided with extensive information about the study, its purpose, design, and what their participation would entail. Participants were informed that participation was voluntary, not compensated, and that they could withdraw at any point. All participants provided informed consent before participation. Data collection was anonymized.

### vCare-PSQ Pilot Study

#### Design and Setting

The pilot study was conducted as a cross-sectional online survey study with a convenience sample of Danish residents who had attended a VC with their GP. Denmark has a government-funded universal health care system which is governed regionally across the 5 regions of Denmark [24]. All Danish citizens and permanent residents are automatically enrolled in the free public health care system and upwards of 98% (approximately 5,850,378) of the population is registered with a GP. The primary care system thus often represents the first point of contact for individual health care needs, with GPs acting as gatekeepers for nearly all secondary and tertiary care. Importantly, GPs operating in the Danish health care system are private business owners who subcontract to the government. As such, the official working agreement between the National Organization for GPs and the Danish government—specifying working conditions and purview, rates and key performance indicators for GPs—is renegotiated and updated at semiregular intervals. Most recently, the agreement was amended in 2022 with the requirement that all GPs in the Danish primary care system make VCs available to their patients by the end of 2024.

#### Sample and Data Collection

The Danish “National Organization of General Practitioners” (in Danish “Praktiserende Lægers Organisation”) recruited 33 GPs to assist with patient recruitment. Participating GPs were from the Regions of Southern Denmark (n=14), Central Denmark Region (n=12), Zealand (n=2), and the Capital (n=5).

A total of 8896 patients were invited by their GP via secure SMS to participate in this study. Invites with a link to the survey were sent via secure SMS from the GP immediately following a completed VC. Data collection took place between April 4, 2022, and August 9, 2022. The survey was coded in a forced-entry format and distributed via SurveyXact (Ramboll). The survey represented a routine monitoring of practice activity and as such no incentive was offered to participants for their participation, nor was there a special focus on enhancing response rates.

#### Analytic Approach

We generated single-item frequencies, means, and SDs, as well as item-rest Pearson correlations, average inter-item correlations, and Cronbach α for the PS-14 validity analysis (aim 1). We then created bar diagrams for patient-reported pros and cons of VC by quartile of patient satisfaction to assess the relevance of contextual variables for satisfaction (aim 2). Finally, we calculated descriptive statistics for general patient satisfaction and the key variables of patient trust, familiarity, IT literacy, and health anxiety, and explored the association between each of these key variables and patient satisfaction in separate linear regression models (aim 3). Specifically, we ran a crude (univariate) model first (model 1), followed by an adjusted model that controlled for covariates of participant age, gender, education, and living status (model 2). In a third model (model 3), we controlled for all key variables and covariates. In addition, we calculated Pearson correlation coefficients for all key variables and patient satisfaction. IBM SPSS Statistics (version 28.0.1.0; IBM Corp) was used for all analyses. The statistical significance level was set at α<.05.

## Results

### Sociodemographics

Of 8896 people invited to participate, a total of 188 completed the survey (response rate: 2.1%). The sample comprised 65.4% (n=123) women. The mean age was 51 (SD 15.27) years. In terms of living status, 23.4% (n=44) reported living alone, while 76.6% (n=144) lived with others (spouse or partner n=120, 64.1%, and “someone else” n=16, 8.5%). Most participants (n=130, 69.2%) were college-educated (bachelor’s degree or higher; see Table 1).

**Table 1:** Participant demographics for age, living status, and education (N=188).

			Total sample		Women (n=123, 65.4%)		Men (n=65, 34.6%)
Age (years), mean (SD)			51.26 (15.27)		49.36 (13.69)		54.85 (17.46)
**Living status, n (%)**							
	Single	44 (23.4)		26 (21.1)		18 (27.7)	
	With others	144 (76.6)		97 (78.9)		47 (72.3)	
**Education, n (%)**							
	Elementary school	14 (7)		8 (6.5)		6 (9.2)	
	Vocational	27 (13.5)		15 (12.2)		12 (18.5)	
	High school	16 (8)		10 (8.1)		6 (9.2)	
	Some college	39 (19.5)		28 (22.8)		11 (16.9)	
	Bachelor’s degree	70 (35)		50 (40.7)		20 (30.8)	
	Master’s degree or higher	21 (10.5)		12 (9.8)		9 (13.8)	

### PS-14 Validity Analysis

Single-item statistics are presented in Table 2. Internal consistency was relatively high with an average item-total correlation of β=0.61 (SD 0.07). As a measure of validation for the adapted PS-14, we calculated Cronbach α statistics. The results indicated high reliability with a Cronbach α of 0.91 for the 14 items.

**Table 2:** Patient satisfaction item results for validation of the patient satisfaction scale (PS-14; average interitem correlation=0.61; Cronbach α=0.91).

Item	n_missing	Mean (SD)	Item-rest correlation
1	0	4.39 (1.04)	0.69
2	0	4.53 (0.80)	0.66
3	0	3.01 (0.98)	0.41
4	0	3.89 (1.26)	0.62
5	0	4.03 (1.06)	0.62
6	0	4.18 (1.20)	0.52
7	0	4.09 (0.97)	0.57
8	0	4.02 (1.05)	0.59
9	0	3.78 (1.15)	0.51
10	0	4.32 (0.86)	0.66
11	0	4.43 (0.75)	0.61
12	0	4.45 (0.86)	0.74
13	5	4.36 (0.90)	0.69
14	5	3.54 (1.13)	0.58

### Patient-Reported Pros and Cons

**Figure 1:**
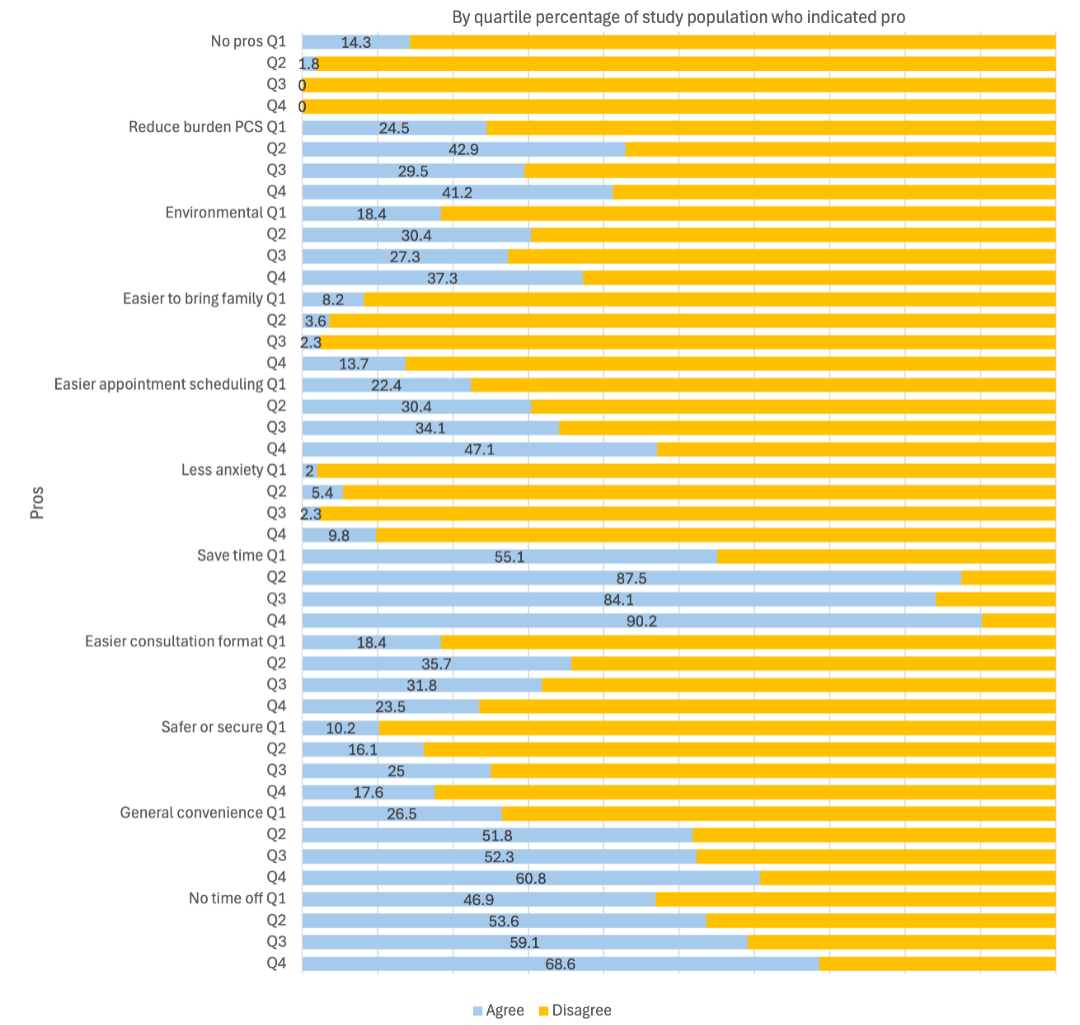
Participant agreement with VC pros by quartile (Qx) of patient satisfaction (Q1=lowest satisfaction and Q4=highest satisfaction). PCS: primary care system; VC: video consultation.

**Figure 2:**
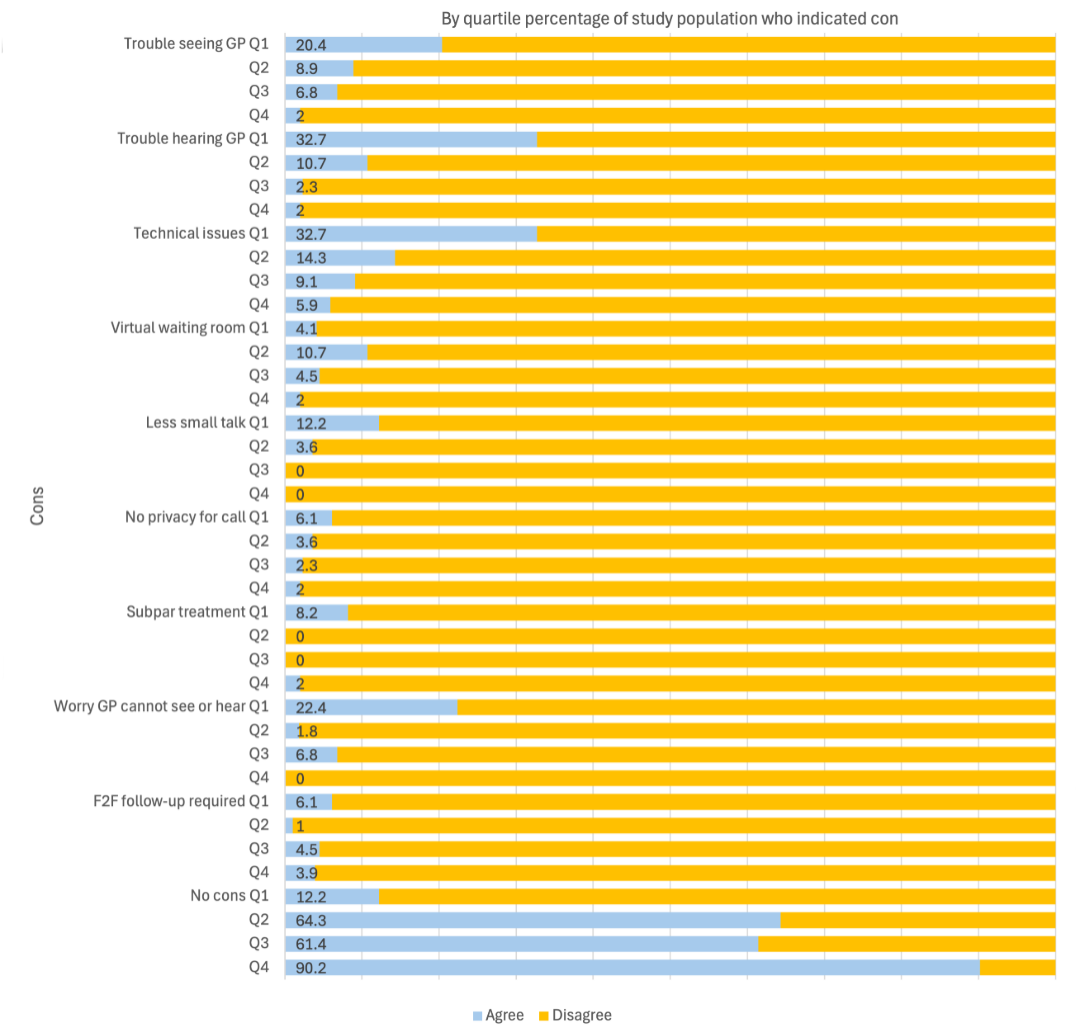
Participant agreement with VC cons by quartile (Qx) of patient satisfaction (Q1=lowest satisfaction and Q4=highest satisfaction). F2F: face-to-face; GP: general practitioner; VC: video consultation.

Figures 1 and 2 show specific pros (Figure 1) and cons (Figure 2) associated with participants’ VC experience and by quartile of patient satisfaction (quartile [Qx]; Q1=lowest satisfaction and Q4=highest satisfaction). Overall, participants perceived considerably more pros than cons, with the most frequently reported pros relating to saving time (n=155, 82.2%), requiring less time off work (n=110, 58.4%), and general convenience (n=94, 50.2%). Environmental sustainability and reducing the burden on the primary care system were also prominent pros. The most common cons concerned general technological issues (n=31, 16.4%), audio issues (n=24, 12.8%), and video issues (n=20, 10.5%).

Further, across quartiles of patient satisfaction, there was a general overall trend (with some variation—eg, concerning “easier to bring family” and “reduce burden on primary care system”) where patients reporting the highest levels of satisfaction (Q4) were more likely to report pros, indicating a positive correlation between patient satisfaction and the experience of pros. This general association was reversed in terms of reported cons across quartiles of patient satisfaction (Figure 2). Patients who were most satisfied also reported fewer cons.

### Patient Satisfaction and Key Variables

Table 3 presents means and SDs for patient satisfaction and each of our key variables of trust in GP, familiarity with GP, IT literacy, and health anxiety. As noted in the introduction, these key variables were selected based on previous evidence of their relevance to patient satisfaction [11,19,20]. Participants scored relatively high on satisfaction, trust, and IT literacy, with each mean score being statistically significantly higher than the given scale midpoint. By contrast, participants scored significantly lower than the scale midpoint for health anxiety in the lead-up to their VC.

**Table 3:** Descriptive statistics for the primary outcome (patient satisfaction) and key variables: scale midpoint, means, SD, minimum, and maximum.

Variable	Scale midpoint	Mean (SD)	Minimum-maximum
Patient satisfaction	3.0	4.08^a^ (0.66)	1.00-5.00
Trust in GP^b^	2.5	3.78^a^ (0.55)	1.00-4.00
Familiarity with GP	2.5	2.66 (1.13)	1.00-4.00
IT literacy	2.5	3.46^a^ (0.80)	1.00-4.00
Health anxiety	3.0	1.94^a^ (1.19)	1.00-5.00

^a^Value different from the scale midpoint at *P*<.001.

^b^GP: general practitioner.

Looking at the correlation between patient satisfaction and our key variables, patient trust in their GP was associated with increased patient satisfaction (β=.50; *P*<.001; 95% CI 0.38 to 0.60) as was familiarity with the GP (β=.17; *P*=.005; 95% CI 0.03 to 0.31) and IT literacy (β=.34; *P*≤.001; 95% CI 0.21 to 0.46). Further, health anxiety was associated with decreased patient satisfaction (β=–.28; *P*=.06; 95% CI –0.43 to –0.13; Table 4).

**Table 4:** Crude (model 1) and adjusted regression models with patient satisfaction as an outcome. Model 2 was adjusted for age, gender, education, and living status. In model 3, all explanatory key variables were included (N=188)^a^.

	Patient satisfaction, *r*	Model 1, β (95% CI)	Model 2, β (95% CI)	Model 3, β (95% CI)
Trust in GP^b^	0.50^c^	.50^c^ (0.43 to 0.72)	.53^c^ (0.45 to 0.74)	.49^c^ (0.36 to 0.68)
IT literacy	0.34^c^	.34^c^ (0.16 to 0.38)	.36^c^ (0.17 to 0.39)	.31^c^ (0.12 to 0.38)
Health anxiety	–0.28^c^	–.28^c^ (–0.24 to –0.07)	–.34^c^ (–0.27 to –0.09)	–.11 (–0.14 to 0.02)
Familiarity GP	0.17^d^	.17^d^ (0.02 to 0.18)	.17^d^ (0.01 to 0.18)	.06 (–0.05 to 0.12)

^a^*P*<.10, * *P*<.05, ** *P*<.01.

^b^GP: general practitioner.

^c^*P*<.01.

^d^*P*<.05.

Results from regression analyses (Table 4) indicated that trust, familiarity, and IT literacy predicted increased patient satisfaction (trust: β=.50; *P*<.001; 95% CI 0.43 to 0.72; familiarity: β=.17; *P*=.01; 95% CI 0.02 to 0.18; IT literacy: β=.34; *P*<.001; 95% CI 0.16 to 0.38, respectively), while health anxiety was associated with decreased satisfaction (β=–.28; *P*<.001; 95% CI –0.24 to –0.07). These effects persisted and were largely unchanged in the adjusted model 2. Controlling for the satisfaction correlates in the fully adjusted model 3, the effects remained stable for trust and IT literacy. However, the association between familiarity and patient satisfaction diminished to the point of statistical nonsignificance (β=.06; *P*=.17; 95% CI –0.05 to 0.12), and the correlation between health anxiety and patient satisfaction decreased in magnitude by over 66% (β=–.11; *P*=.06; 95% CI –0.14 to 0.02).

## Discussion

### Principal Findings

In this study, we set out to develop and pilot a comprehensive measure of patient satisfaction with remote care delivery, integrating a standardized assessment of satisfaction with both situational and patient-doctor relational factors. The multidimensional structure of our questionnaire performed well in a pilot sample, with our results largely showing the expected patterns in patient satisfaction and the included correlates. Regarding our results for aim 1, the adaptation of the PS-14—the core, static part of the vCare-PSQ—to a Danish general population setting exhibited high internal consistency. This scale thus represents a viable measure of patient satisfaction with VC in this population. Given the general scarcity of standardized scales of this kind at a time when VC is becoming exceedingly more common, the preliminary validation of the PS-14 is not only timely but also necessary for future studies into VC feasibility and implementation. We recommend the use of this scale in future research as a fixed measure of patient satisfaction with VC that can be compared across time, place, and setting. Importantly, however, we also note the need for further reliability and validity testing of this scale with more diverse and representative study populations.

In terms of aim 2 and as expected, the addition of the patient-reported pros and cons added extra insight into the correlates of the PS-14. For our sample, general convenience and saving time were the most common self-reported reasons for patient satisfaction, confirming existing studies in this area [2-7]. These were followed by the perception that VC may alleviate an overburdened primary care system and offset the environmental impact of commuting to and from the GP. This likely reflects an awareness in the general population of several issues, primarily related to funding and staff shortages, that encumber the Danish health care system. Participants reported very few cons overall, most of which related to technology issues. Further, many of the cons reported in other studies (eg, medical mistrust, privacy issues, or subpar treatment) were virtually nonexistent in our sample. Beyond these descriptive results, however, the observed variation in terms of which pros and cons were reported by patients provided more detailed knowledge about why patients were satisfied or not with VC. Further, breaking this data down by quartiles of patient satisfaction also revealed a consistent correlation with the PS-14, indicating the value of this data as the dynamic context within which the observed level of patient satisfaction emerges.

Finally, examining the significance of the other more tacit factors included in aim 3, we also found that patient trust, familiarity with their GP, health anxiety, and IT literacy may feed into the patient experience of VC to increase satisfaction. We note that these associations were detectable even in our small, self-selected sample and persisted when controlling for relevant sociodemographic factors. Specifically, our results suggested the importance of IT literacy for patient satisfaction. This finding aligns with numerous other studies on this topic and emphasizes the somewhat obvious point that VC implementation and use is in large part contingent on patients’ IT skills [25,26]. Further, and as expected, health anxiety correlated negatively with patient satisfaction. This may reflect constraints imposed by VC on the subtle communication of empathy, reassurance, and “bedside manner” which may help quell anxiety in face-to-face interactions [27]. Finally, trust emerged as a key factor that appeared to drive patient satisfaction with VC more than any other variable included in our analyses (the effect for familiarity decreased considerably in magnitude once trust was accounted for). We interpret these findings as an indication of the crucial importance of the relational aspects of health care delivery.

In sum, supplementing a standardized and one-dimensional patient-satisfaction questionnaire (the PS-14) with additional items, which tapped both situational variables (aim 2) as well as intrinsic and relational factors (aim 3), appeared to enhance the comprehensiveness of the PS-14 to include a wider range of relevant factors that feed into patient satisfaction with VC.

### Implications and Future Directions

Our results have several empirical and practical implications. A key contribution of our study relates to the evidence-based development and validation of a quantitative patient satisfaction measurement tool. As noted in the introduction, much of the knowledge on patient satisfaction with VC is based on studies with relatively narrow definitions and operationalizations of satisfaction. We argue that optimal VC implementation and acceptability are contingent on the extent to which patients perceive this format of health care delivery as useful and satisfactory. To this end, the vCare-PSQ format may provide valuable insight into the multidimensional variables that shape patient satisfaction. Specifically, acknowledging the multifaceted and fluid nature of patient satisfaction, a key contribution of this study relates to the conceptualization of patient satisfaction in terms of a three-dimensional structure. We operationalize this structure by combining a standardized and readily comparable measure of patient satisfaction (the PS-14) with both relational and dynamic setting-specific components into a single measurement instrument (the vCare-PSQ) that taps the broader context in which satisfaction or dissatisfaction arises in a given population. In addition, the fact that the dynamic aspects of the vCare-PSQ can (and probably should) be tailored to the specific context in which it is implemented, makes this questionnaire adaptable to multiple settings. Indeed, we encourage future studies to use the vCare-PSQ as a starting point from which to develop questionnaires that are more specific to a given population, time, or setting.

### Strengths and Limitations

Our study had several notable strengths. These included primarily the rigorous survey development methodology that comprised a full literature review of past questionnaires, cognitive interviews with patients, individual semistructured interviews with patients, and extensive testing by experts in primary and digital care. However, there are also limitations that should be noted. Importantly, with an exceedingly low response rate of 2.11% (N=188), our results are based on a small, self-selected sample of the target population. This fact alone raises important concerns about the external validity of our results. Further to this point, as we had no access to sociodemographic data for those individuals who chose to not participate in this study, we have no way of assessing whether and how our sample diverges from the source population. There are, however, differences between our sample and the general adult Danish population, including an overrepresentation of women and people with high SES (as indicated by education and IT literacy). This represents a serious limitation as people with low SES and education represent the segment of the population that may benefit the most from remote care options, but also experience the most significant access barriers. Further to this point, we did not have any data on participant race, ethnicity, nationality, or culture. This is important as past research has shown that minoritized populations often are grossly underserved by health care in general as well as telehealth initiatives specifically [15-17,28-30].

### Conclusion

We conducted a study designed to develop a comprehensive questionnaire for the assessment of patient satisfaction with VC. To this end, we pursued 3 aims that included a preliminary validation of an existing patients-satisfaction scale (the PS-14), as well as the identification of additional individual, relational, and situational factors that might provide clearer and more inclusive insight into the drivers of patient satisfaction. These findings fed into the development of the vCare-PSQ. Given the very low response rate and the sociodemographic differences between our study sample and the general Danish population, however, we implore the reader to interpret these findings with caution. Nonetheless, our results are consistent with past research that emphasizes these factors as fundamental determinants of how patients perceive and experience remote consultations such as VCs. This lends credence to the validity and appropriateness of the vCare-PSQ format, which includes not only a static measure of patient satisfaction, but also dynamic contextual and relational variables. We hope that this questionnaire can be adapted and used to monitor and evaluate patient satisfaction with VC in a variety of populations and settings.

## Abbreviations

COSMIN: Consensus-Based Standards for the Selection of Health Measurement Instruments
GP: general practitioner
PS-14: patient-satisfaction scale
VC: video consultation
vCare-PSQ: vCare Patient-Satisfaction Questionnaire

## Data Availability

The anonymized data sets generated or analyzed during this study are available from the corresponding author upon reasonable request.

## References

[1] Assing Hvidt E, Atherton H, Keuper J, Kristiansen E, Lüchau EC, Lønnebakke Norberg B, Steinhäuser J, van den Heuvel J, van Tuyl L (2023). Low adoption of video consultations in post-COVID-19 general practice in northern Europe: barriers to use and potential action points. J Med Internet Res.

[2] Thiyagarajan A, Grant C, Griffiths F, Atherton H (2020). Exploring patients' and clinicians' experiences of video consultations in primary care: a systematic scoping review. BJGP Open.

[3] Anderson J, Walsh J, Anderson M, Burnley R (2021). Patient satisfaction with remote consultations in a primary care setting. Cureus.

[4] Haun MW, Oeljeklaus L, Hoffmann M, Tönnies J, Wensing M, Szecsenyi J, Peters-Klimm F, Krisam R, Kronsteiner D, Hartmann M, Friederich HC (2023). Primary care patients' experiences of video consultations for depression and anxiety: a qualitative interview study embedded in a randomized feasibility trial. BMC Health Serv Res.

[5] Vosburg RW, Robinson KA (2022). Telemedicine in primary care during the COVID-19 pandemic: provider and patient satisfaction examined. Telemed e-Health.

[6] Li HL, Chan YC, Huang JX, Cheng SW (2020). Pilot study using telemedicine video consultation for vascular patients' care during the COVID-19 period. Ann Vasc Surg.

[7] Wanderås MR, Abildsnes E, Thygesen E, Martinez SG (2023). Video consultation in general practice: a scoping review on use, experiences, and clinical decisions. BMC Health Serv Res.

[8] Donaghy E, Atherton H, Hammersley V, McNeilly H, Bikker A, Robbins L, Campbell J, McKinstry B (2019). Acceptability, benefits, and challenges of video consulting: a qualitative study in primary care. Br J Gen Pract.

[9] Assing Hvidt E, Christensen NP, Grønning A, Jepsen C, Lüchau EC (2022). What are patients' first-time experiences with video consulting? A qualitative interview study in Danish general practice in times of COVID-19. BMJ Open.

[10] Jiménez-Rodríguez D, Santillán García A, Montoro Robles J, Rodríguez Salvador MDM, Muñoz Ronda FJ, Arrogante O (2020). Increase in video consultations during the COVID-19 pandemic: healthcare professionals' perceptions about their implementation and adequate management. Int J Environ Res Public Health.

[11] Orrange S, Patel A, Mack WJ, Cassetta J (2021). Patient satisfaction and trust in telemedicine during the COVID-19 pandemic: retrospective observational study. JMIR Hum Factors.

[12] Barkai G, Gadot M, Amir H, Menashe M, Shvimer-Rothschild L, Zimlichman E (2021). Patient and clinician experience with a rapidly implemented large-scale video consultation program during COVID-19. Int J Qual Health Care.

[13] Tsampras N, Craciunas L, Dearden M, Sood A, Mathur R (2023). Video consultations in reproductive medicine: safety, feasibility and patient satisfaction. Eur J Obstet Gynecol Reprod Biol.

[14] Duffy LV, Evans R, Bennett V, Hady JM, Palaniappan P (2023). Therapeutic relational connection in telehealth: concept analysis. J Med Internet Res.

[15] Zhai Y (2020). A call for addressing barriers to telemedicine: health disparities during the COVID-19 pandemic. Psychother Psychosom.

[16] Haynes N, Ezekwesili A, Nunes K, Gumbs E, Haynes M, Swain J (2021). "Can you see my screen?" Addressing racial and ethnic disparities in telehealth. Curr Cardiovasc Risk Rep.

[17] Rivera V, Aldridge MD, Ornstein K, Moody KA, Chun A (2021). Racial and socioeconomic disparities in access to telehealth. J Am Geriatr Soc.

[18] Mekhjian H, Turner JW, Gailiun M, McCain TA (1999). Patient satisfaction with telemedicine in a prison environment. J Telemed Telecare.

[19] Murphy M, Salisbury C (2020). Relational continuity and patients' perception of GP trust and respect: a qualitative study. Br J Gen Pract.

[20] Van Den Assem B, Dulewicz V (2014). Patient satisfaction and GP trustworthiness, practice orientation and performance: implications for selection, training and revalidation. J Health Organ Manag.

[21] Gagnier JJ, Lai J, Mokkink LB, Terwee CB (2021). COSMIN reporting guideline for studies on measurement properties of patient-reported outcome measures. Qual Life Res.

[22] Hammersley V, Donaghy E, Parker R, McNeilly H, Atherton H, Bikker A, Campbell J, McKinstry B (2019). Comparing the content and quality of video, telephone, and face-to-face consultations: a non-randomised, quasi-experimental, exploratory study in UK primary care. Br J Gen Pract.

[23] Robine J, Jagger C (2003). Jagger, Translation & linguistic evaluation protocol & supporting material. WHO/UNESCAP Project on Health and Disability Statistics.

[24] Henriksen DP, Rasmussen L, Hansen MR, Hallas J, Pottegård A (2015). Comparison of the five Danish regions regarding demographic characteristics, healthcare utilization, and medication use—a descriptive cross-sectional study. PLoS One.

[25] Anthony Jnr B (2021). Implications of telehealth and digital care solutions during COVID-19 pandemic: a qualitative literature review. Inform Health Soc Care.

[26] Slattery B, Ackerman L, Jagadamma KC (2022). Service evaluation of telehealth in a physiotherapy musculoskeletal setting: patient outcomes and results from risk stratification. Musculoskeletal Care.

[27] McConnochie KM (2019). Webside manner: a key to high-quality primary care telemedicine for all. Telemed e-Health.

[28] Darrat I, Tam S, Boulis M, Williams AM (2021). Socioeconomic disparities in patient use of telehealth during the coronavirus disease 2019 surge. JAMA Otolaryngol Head Neck Surg.

[29] Rodriguez JA, Saadi A, Schwamm LH, Bates DW, Samal L (2021). Disparities in telehealth use among California patients with limited English proficiency. Health Aff (Millwood).

[30] Zhang D, Shi L, Han X, Li Y, Jalajel NA, Patel S, Chen Z, Chen L, Wen M, Li H, Chen B, Li J, Su D (2024). Disparities in telehealth utilization during the COVID-19 pandemic: findings from a nationally representative survey in the United States. J Telemed Telecare.

